# Comparison of orientation encoding across layers within single columns of primate V1 revealed by high-density recordings

**DOI:** 10.3389/fncir.2024.1399571

**Published:** 2024-09-23

**Authors:** Shude Zhu, Ruobing Xia, Xiaomo Chen, Tirin Moore

**Affiliations:** ^1^Department of Neurobiology and Howard Hughes Medical Institute, Stanford University School of Medicine, Stanford, CA, United States; ^2^Department of Neurobiology, Physiology, and Behavior, Center for Neuroscience, UC Davis, Davis, CA, United States

**Keywords:** neocortical circuitry, laminar organization, orientation decoding, cortical column, high-density recording

## Abstract

Primary visual cortex (V1) has been the focus of extensive neurophysiological investigations, with its laminar organization serving as a crucial model for understanding the functional logic of neocortical microcircuits. Utilizing newly developed high-density, Neuropixels probes, we measured visual responses from large populations of simultaneously recorded neurons distributed across layers of macaque V1. Within single recordings, myriad differences in the functional properties of neuronal subpopulations could be observed. Notably, while standard measurements of orientation selectivity showed only minor differences between laminar compartments, decoding stimulus orientation from layer 4C responses outperformed both superficial and deep layers within the same cortical column. The superior orientation discrimination within layer 4C was associated with greater response reliability of individual neurons rather than lower correlated activity within neuronal populations. Our results underscore the efficacy of high-density electrophysiology in revealing the functional organization and network properties of neocortical microcircuits within single experiments.

## Introduction

Early neurophysiological investigations of primary visual cortex (V1) identified the striking emergence of shape processing by orientation-selective neocortical neurons, as observed first in cats (Hubel and Wiesel, 1962) and subsequently in primates (Hubel and Wiesel, 1968). Input from the dorsal lateral geniculate nucleus (dLGN) is fundamentally transformed in V1 from circular, center-surround receptive fields (RFs) into selectivity for orientation in simple cells. A vast number of past studies have examined the distribution of orientation and other functional properties of V1 neurons across cortical layers in an effort to fully understand the transformation of visual information carried out at this crucial stage of visual processing (Schiller et al., 1976; Poggio et al., 1977; Bauer et al., 1980; Hawken and Parker, 1984; Livingstone and Hubel, 1984; Ringach et al., 1997; Ringach et al., 2002; Gur et al., 2005; Martinez et al., 2002; Niell and Stryker, 2008; Wang et al., 2020). To date, although much is understood about the functional organization and microcircuitry of primate V1, a number of key questions remain unresolved. For example, contrary to early evidence of a lack of orientation selectivity in V1 input layers (4Cα and 4Cβ) (Hubel and Wiesel, 1968), a number of other studies demonstrated that orientation selectivity is more broadly distributed across layers (Schiller et al., 1976; Hawken and Parker, 1984; Ringach et al., 2002; Martinez et al., 2002; Gur et al., 2005; Niell and Stryker, 2008; Wang et al., 2020). Although a wealth of computational models has been proposed to explain the emergence of orientation selectivity in V1 (e.g., Adorjan et al., 1999; McLaughlin et al., 2000; Priebe and Ferster, 2012; Goris et al., 2015; Chariker et al., 2016), the validity of such models rests on the availability of sufficient data to test key predictions and assumptions.

Historically, the bulk of neurophysiological measurements of the visual properties of macaque V1 neurons have been carried out in successive extracellular recordings from individual neurons or small numbers of neurons using conventional single-electrodes (e.g., Schiller et al., 1976; Hawken and Parker, 1984; Livingstone and Hubel, 1984; Ringach et al., 1997) or low-channel count linear arrays (Hansen et al., 2012; Nigam et al., 2019; Ziemba et al., 2019). Typically, from such data, the distributions of those properties are studied in aggregated sets of recordings accumulated across multiple sessions. As a result, direct comparisons between subpopulations of neurons within local circuits, e.g., within single cortical columns, are less than ideal. Recent advances in recording technology have facilitated the development of high-density micro-electrode arrays resulting in a substantial increment (~20x) in the number of neurons that can be studied simultaneously within a localized area of neural tissue. A prime example is the recent development of the Neuropixels probe (IMEC, Inc.), which consists of a high-channel count Si shank with continuous, dense, programmable recording sites (~1,000/cm). Numerous recent studies have demonstrated the advantages of such probes, such as their use in recording large neuronal populations within deep structures where optical approaches cannot be deployed (Jun et al., 2017; Steinmetz et al., 2019). However, only a few electrophysiological studies of the primate brain have been carried out thus far (Trautmann et al., 2019; Hesse and Tsao, 2020; Trepka et al., 2022; Sun et al., 2022; Trautmann et al., 2023; Zhang et al., 2024).

Using Neuropixels probes, we studied the visual activity of populations of neurons distributed across layers of anesthetized macaque V1. The large capacity of Neuropixels probes facilitated comparisons between substantial populations of neurons within single cortical columns, both within and between defined laminar compartments. Robust differences in the functional properties of neurons within different subpopulations were observable within a single recording session. Most surprisingly, although standard measurements of orientation selectivity yielded only minor differences between laminar compartments, we found that decoding of orientation from layer 4C neuronal responses outperformed superficial and deep layer neurons within the same cortical column. Furthermore, the superior orientation decoding from layer 4C activity was associated with greater response reliability of individual neurons rather than from the lower correlated activity among layer 4C neurons. The results demonstrate the utility of high-density electrophysiology in revealing the functional organization of primate neocortical microcircuits in single experiments.

## Methods

### Lead contact and materials availability

Further information and requests for resources and reagents should be directed to and will be fulfilled by the Lead Contact, Shude Zhu (shude@stanford.edu).

### Experimental model and subject details

Anesthetized recordings were conducted in 2 adult male Rhesus macaques (*Macaca Mulatta*, M1, 13 kg; M2 8 kg). The number of animals used is typical for primate neurophysiological experiments. All experimental procedures were in accordance with National Institutes of Health Guide for the Care and Use of Laboratory Animals, the Society for Neuroscience Guidelines and Policies, and with approved Institutional Animal Care and Use Committee (IACUC) protocol (#APLAC-9900) of Stanford University.

### Method details

#### Electrophysiological recordings

Prior to each recording session, treatment with dexamethasone phosphate (2 mg per 24 h) was instituted 24 h to reduce cerebral edema. After administration of ketamine HCl (10 mg per kilogram body weight, intramuscularly), monkeys were ventilated with 1–2% isoflurane in a 1:1 mixture of N_2_O and O_2_ to maintain general anesthesia. Electrocardiogram, respiratory rate, body temperature, blood oxygenation, end-tidal CO_2_, urine output and inspired/expired concentrations of anesthetic gases were monitored continuously. Normal saline was given intravenously at a variable rate to maintain adequate urine output. After a cycloplegic agent (atropine sulfate, 1%) was administered, the eyes were focused with contact lenses on an LCD monitor. Vecuronium bromide (60 μg/kg/h) was infused to prevent eye movements.

With the anesthetized monkey in the stereotaxic frame, an occipital craniotomy was performed over the opercular surface of V1. The dura was reflected to expose a small (~3 mm^2^) patch of cortex. Next, a region relatively devoid of large surface vessels was selected for implantation, and the Neuropixels probe was inserted with the aid of a surgical microscope. Given the width of the probe (70 um x 20 um), insertion of it into the cortex sometimes required multiple attempts if it flexed upon contacting the pia. The junction of the probe tip and the pia could be visualized via the (Zeiss) surgical scope, and the relaxation of pia dimpling was used to indicate penetration, after which the probe was lowered at least 3–4 mm. Prior to probe insertion, it was dipped in a solution of the DiI derivative FM1-43FX (Molecular Probes, Inc) for subsequent histological visualization of the electrode track.

Given the length of the probe (1 cm), and the complete distribution of electrode contacts throughout its length, recordings could be made either in the opercular surface cortex (M1) or within the underlying calcarine sulcus (M2), by selecting a subset of contiguous set of active contacts (*n* = 384) from the total number (*n* = 986). Receptive fields (RFs) from online multi-unit activity were localized on the display using at least one eye. RF eccentricities were ~ 4–6° (M1) and ~ 6–10° (M2). Recordings were made at 1 to 3 sites in one hemisphere of each monkey. At the end of the experiment, monkeys were euthanized with pentobarbital (150 mg kg^−1^) and perfused with normal saline followed by 1 liter of 1% (wt/vol) paraformaldehyde in 0.1 M phosphate buffer, pH 7.4.

#### Visual stimulation

Visual stimuli were presented on an LCD monitor (NEC-4010; dimensions: 88.5 cm H* 49.7 cm V; resolution: 1360 * 768 pixels; frame rate: 60 Hz) positioned 114 cm from the monkey. The stimuli consisted of drifting Gabor gratings (2 deg./s., 100% Michelson contrast) with a diameter of 1.5 degrees of visual angle (dva), positioned within the joint receptive fields (RFs) of recorded neurons. This size was selected to largely constrain the stimuli within the RFs of recorded neurons, typically ~0.5–1 dva as determined manually during experiments. Gratings drifted in 36 different directions between 0 to 360° in 10° steps in a pseudorandom order. The stimulus in each condition was presented for 1 s and repeated 5 or 10 times. A blank screen with equal luminance to the Gabor patch was presented for 0.25 s during the stimulus interval. Stimuli were presented either monocularly (sessions 1 and 4) or both monocularly and binocularly (sessions 2,3, and 5). Four spatial frequencies (0.5, 1, 2, 4 cycles/deg.) were tested. The optimal eye and spatial frequency conditions were determined offline for further analysis.

#### Layer assignment

The laminar location of our recording sites was estimated based on a combination of functional analysis and histology results. For each recording, we first performed the current source density (CSD) analysis on the stimulus-triggered average of local field potentials (LFP). LFP were low-pass filtered at 200 Hz and recorded at 2500 Hz. LFP signals recorded from each 4 neighboring channels were averaged and realigned to the onset of visual stimulus. CSD was estimated as the second-order derivatives of signals along the probe axis using the common five-point formula (Nicholson and Freeman, 1975). The result was then smoothed across space (*σ* = 120 μm) to reduce the artifact caused by varied electrode impedance. We located the lower boundary of the major sink (the reversal point of sink and source) as the border between layer 4C and layer 5/6. We also considered anatomical data in order to localize recorded neurons within gray matter, allowing for minor adjustments (± 1 group channel) in layer boundary placement. Subsequent layer boundaries were determined by offsetting the cortical thickness from histological images. For example, the boundary between layers 4A/B and layer 4C was determined by offsetting the thickness of layer 4C in the histology image.

#### Data acquisition and spike sorting

Raw spike-band data was sampled and recorded at 30 kHz. It was then median-subtracted and high-pass filtered at 300 Hz during the pre-processing stage. Spike-sorting was carried out with Kilosort2 (Pachitariu et al., 2024) to find spike times and assign each spike to different units. The raw sorted data was then manually curated in Phy^1^ to remove spikes with atypical waveforms and perform minimal merging and splitting. Some key parameters in Kilosort2 that we used: Ops.th = [10,4]; Ops.lam = 20; Ops.AUCsplit = 0.9; Ops.ThPre = 8; Ops.spkTh = −6.

#### Single neuron properties

To characterize neuronal properties, the evoked activity was assessed using mean firing rate (spikes/sec) over the whole stimulus presentation period, offset by response latency delay. Only responses to the preferred eye and spatial frequency were selected for further analysis. The maximum firing rate was the neuron’s response to the preferred drifting orientation and direction. Modulation ratio was defined as F1/F0, where F1 and F0 are the amplitude of the first harmonic at the temporal frequency of drifting grating and constant component of the Fourier spectrum to the neuron’s response to preferred orientation. Direction selectivity (Direction Index, DI) was determined as the response to preferred orientation and drift direction minus the response to preferred orientation but opposite drift direction, divided by the sum of these two responses (Swindale, 1998). Orientation selectivity (Orientation Index, OI) was determined as the response to preferred orientation minus the response to orthogonal orientation, divided by the sum of these two responses (Swindale, 1998). To estimate the orientation tuning bandwidth, the orientation tuning responses were first smoothed with a Hanning window (half width at half height of 20^°^), and then fitted with a von-Mises function (Swindale, 1998)


$$y=a_{0}+a_{1}*e^{a_{2}*\left(\cos\left(2*x-2*a_{3}\right)-1\right)}$$

Only neurons that were well fit by the function (R^2^ > 0.7) were included in the bandwidth analysis. The locations of the peak of the fitted curves were determined. The two orientations closest to the peak on either side of the tuning curve where responses dropped to $1/\sqrt{2}$ of the peak response were then estimated (Schiller et al., 1976). Bandwidth was defined as the half of the differences between the two orientations. If the response around the peak never went below the response criteria, the tuning bandwidth was defined as 180^°^.

Fano factor was computed to assess individual neuron’s response variability. For each stimulus condition, spikes events were counted in a 100 ms window for all the trial repetitions. And Fano factor were defined as the spike counts variance across trial divided by spike counts mean. And those ratios were then averaged across the whole stimulus presentation period and across all stimulus conditions to generate the Fano factor for that neuron.

#### Machine learning algorithms

Five widely used machine learning algorithms were employed to evaluate the performance of neuronal populations in encoding stimulus orientations. The algorithms used were Logistic Regression (LogR), Linear Discriminant Analysis (LDA), Gaussian Naïve Bayes (NB), Support Vector Machines (SVM) and Random Forest (RF). These algorithms were chosen to corroborate decoding results using complementary schemes, and to ensure that comparisons of decoding performance across different layers were not biased by features uniquely detected by specific algorithms.

Logistic Regression (LogR) is a probabilistic linear classifier commonly used to discriminate binary classes. It assumes that the probability of a class can be expressed as a log-linear transformation of the input features. Given a neuronal population response vector ***X*** = (*x_1_, …, x_N_*) on a single trial, where *x_i_* represents number of spikes fired by neuron *i* and *N* is the total number of features/neurons in the population, the conditional probability that this population response vector occurred in a trial with stimulus class *k* ∈{0, 1} is given by:


$$P\left(y=k|X\right)=\sigma \left(\omega_{k}^{T}X+b_{k}\right)$$

Here, *σ*(z) = 1/(1 + exp.(−z)) is the logistic function, which maps real-valued numbers to the range (0,1) to represent probabilities. ***X*** is the input feature vector (population response with dimension *N* x 1). $\omega_{k}$ is the vector of decoding weights for each neuron to class *k*, with the same dimension as ***X***. *b_k_* is the bias (intercept) term. The model parameters of weights and bias are fitted using maximum likelihood estimation. The predicted class is chosen as the one that maximizes the posterior probability. To prevent overfitting, we trained the model with L_2_ regularization.

Linear Discriminant Analysis (LDA) is also a probability-based linear model, but additionally assumes that the input features follow a multivariant Gaussian distribution within each class, and that all classes share the same covariance matrix. The logarithm of the posterior probability that a neuronal population response vector ***X*** belongs to stimulus class k is given by:


$$\mathrm{logP}\left(y=k|X\right)=\omega_{k}^{T}X+b_{k}$$

The first term represents the linear combination of the features, where ***X*** is the input feature vector and $\omega_{k}=\Sigma^{-1}\mu_{k}$ is the decoding weight vector for class k. Σ is the shared covariance matrix across all classes, and $\mu_{k}$ is the mean vector of the input features for class *k*. The second term, $b_{k}=-\frac{1}{2}\mu_{k}^{T}\Sigma^{-1}\mu_{k}+\mathrm{logP}\left(y=k\right)$ , is a constant bias incorporating the prior probability (the relative frequency) for each class. By estimating the covariance matrix from the entire training dataset, LDA is generally considered robust and can perform well in situations where there may not be enough trials to accurately estimate covariance matrices for individual classes (Bishop, 1995; Averbeck et al., 2003).

Gaussian Naïve Bayes (NB) algorithm is another probabilistic classifier yet differs significantly from LDA in its assumptions and approach. Gaussian NB assumes that input features are normally distributed, but assumes conditional independence between features given the class label. Specifically, each feature is modeled as a Gaussian distribution with class-specific mean and variance, resulting in a generally nonlinear decision boundary. This independence assumption implies that the covariance between features is zero within each class, making Gaussian NB a correlation-blind classifier. Consequently, it is often used to assess the contribution of spike-count correlations (noise correlations) between neurons. Given a neuronal population response vector ***X*** = (*x_1_, …, x_N_*) on a single trial, where *x_i_* represents number of spikes fired by neuron *i* and *N* is the total number of features/neurons in the population, the decoded stimulus class $\hat{y}$ can be determined as the class *k* ∈{0, 1} that maximizes the following posterior function:


$$\hat{y}=\arg\max_{k\in \left\{0,1\right\}}P\left(y=k\right)\overset{n}{\underset{i=1}{\prod }}P\left(x_{i}|y=k\right)$$

Here, $P\left(y=k\right)$ represents the prior probability of each class, which reflects the relative frequency of each class. The likelihood of each feature *x_i_* given the class *y* = *k* is modeled as:


$$P\left(x_{i}|y=k\right)=\frac{1}{\sqrt{2\pi \sigma_{ik}^{2}}}\exp\left(-\frac{{\left(x_{i}-\mu_{ik}\right)}^{2}}{2\sigma_{ik}^{2}}\right)$$

Under the conditional independence assumption, the joint distribution of the population response given a stimulus class *k* is the product of individual feature distribution $P\left(x_{i}|y=k\right)$ . Here, *μ_ik_* is the mean response of neuron *i* to stimulus class *k,* and *σ_ik_^2^* is the noise variance.

Support Vector Machine (SVM) aims to find a hyperplane in a high-dimensional space that maximizes the margin, which is the distance between the hyperplane and the nearest training samples from any class. Unlike algorithms that rely on specific distributional assumptions, SVM learns the underlying structures of the neuronal response distributions directly from the data. In the case of a soft-margin SVM, the following primal optimization problem is solved:


$$\min_{\omega ,b,\xi \;}\frac{1}{2}\omega^{T}\omega +C\sum \xi_{s}$$

$$\mathrm{subject}\;to\;y_{s}\left(\omega^{T}\Phi \left(X_{s}\right)+b\right)\geq 1-\xi_{s}\;\mathrm{and}\;\xi_{s}\geq 0,for\;\mathrm{each}\;\mathrm{sample}\;s$$

Here, for each training sample *s* (representing individual trials), *y_s_* is the class label (either 1 or − 1), ***X***_***s***_ is the input feature vector, **w** is the weight vector (normal to the hyperplane), and *b* is the bias (intercept). The term $\omega^{T}\omega$ represents the inverse of the margin. $\xi_{s}$ is a slack variable that allows for some misclassifications. The primary objective of the SVM model is to maximize the margin by minimizing $\omega^{T}\omega$ , ensuring that the decision function $\omega^{T}\Phi \left(X_{s}\right)+b$ returns positive values for samples belonging to class 1 and negative values for samples belonging to class −1. To handle cases where the data is not perfectly separable by a hyperplane, the model permits some samples to lie within a distance $\xi_{s}$ from their correct boundary. The regularization parameter *C* controls the trade-off between maximizing the margin and minimizing classification error. The radial basis function (RBF) kernel was employed in this study to project the dataset into a higher-dimensional space.

Random Forest algorithm is an ensemble learning method that constructs multiple randomized decision trees to improve model performance. A decision tree is a non-parametric supervised learning method that predicts the value of a target variable by applying simple decision rules inferred from input features. In Random Forest, the algorithm creates multiple subsets of the original training dataset by randomly sampling with replacement. During the construction of each tree, when splitting a node, the algorithm searches for the best split among a random subset of features (Ho, 1995). These randomization processes result in an ensemble of decision trees, with the final prediction determined by averaging the probabilistic predictions from the individual trees. By introducing more diversity among the individual trees in the ensemble, Random Forest reduces the risk of overfitting and enhances generalization. Additionally, it effectively captures complex, nonlinear relationships between features and the target variable.

#### Decoding analysis

Decoding analyses were performed using Python’s scikit-learn package, employing built-in functions of “LogisticRegressionCV,” “LinearDiscriminantAnalysis,” “GaussianNB,” “SVC,” “RandomForestClassifier,” respectively (Pedregosa et al., 2011). For each recording session, binary decoders were constructed from each neuronal subpopulation’s responses to discriminate between pairs of stimulus orientations. All pairwise combinations of the 18 tested orientations were used, resulting in 153 orientation pairs to discriminate. Neuronal subpopulations consisted of a fixed number of 10 adjacent single neurons, with the depth of the neurons at the center determining the assignment of this subpopulation to the corresponding laminar compartment. Responses were calculated as the mean spike counts within the visual stimulation period (50 ms to 1,050 ms after stimulus onset, accounting for response latency), and were normalized by the maximum value for each neuron. Only trials tested with gratings of the optimal spatial frequency for the subpopulation were selected, resulting in 20–40 trials per orientation pair.

For each of the five algorithms, a 10-fold cross-validation procedure was performed. The dataset was randomly split into 10 folds, with each fold used for testing once and the remaining 9-folds for training. During training, for logistic regression, the models were regularized with L_2_ penalty terms, and the optimal regularization parameter *C* was evaluated within the range of [10^−6^, 10^−5^, 10^−4^, 10^−3^, 10^−2^, 10^−1^, 10^0^, 10^1^, 10^2^, 10^3^, 10^4^] using 6-fold validation on the training dataset. For LDA, the “lsqr” solver was chosen for the least square solution, with the shrinkage option set to “auto.” Gaussian NB and Random Forest classifiers were used with default parameters. For SVM, the “rbf” kernel was used and the optimal regularization parameter *C* was evaluated within the range of [10^−6^, 10^−5^, 10^−4^, 10^−3^, 10^−2^, 10^−1^, 10^0^, 10^1^, 10^2^, 10^3^, 10^4^] using 6-fold validation on the training dataset. Decoding performance was defined as the percentage of correct classifications on the test dataset. To compare the performance of different subpopulation within different laminar compartments, decoding accuracy across different orientation pairs was averaged.

#### Neuronal dropping curves

Instead of a fixed number of 10 neurons, NDCs were generated using population decoders with varied subpopulation size. For each recording session, neurons were systematically selected in increasing numbers (*n* = 1, 2, …) within each laminar compartment to discriminate all 153 pairwise orientation combinations. Each selection for a given *n* consisted of different combinations of neurons, repeated up to 200 times. LDA classifier was chosen for this analysis due to its similar performance compared to other classifiers in the 10-neuron subpopulation decoding, and its computational efficiency and robustness (Bishop, 1995).

#### Decoding sensitivity

Decoding sensitivity was determined for each neuronal subpopulation. For each orientation, decoder performance in discriminating orientation pairs was expressed as a function of the change in orientation (Δθ = 10°, 20°, …, 90°) relative to the reference orientation. The minimum difference (Δθ_min_) was interpolated so that the interpolated decoder performance reached a threshold level of 60%. Sensitivity was calculated as the inverse of Δθ_min_. Thus, sensitivity measures how small the differences in orientation can be for the decoder to achieve 60% accuracy, with higher sensitivity indicating the ability to robustly discriminate pairs with smaller differences.

#### Shuffled population and single neuron decoders

Shuffled population and single neuron decoders were constructed similarly, with modifications. For shuffled decoders, trials of each orientation for each neuron in the 10-neuron subpopulations were independently shuffled in the training dataset, and performance was tested on un-shuffled test dataset. For single neuron decoder, only individual neuron was used instead of 10 adjacent neuronal subpopulations.

## Results

**Figure 1:**
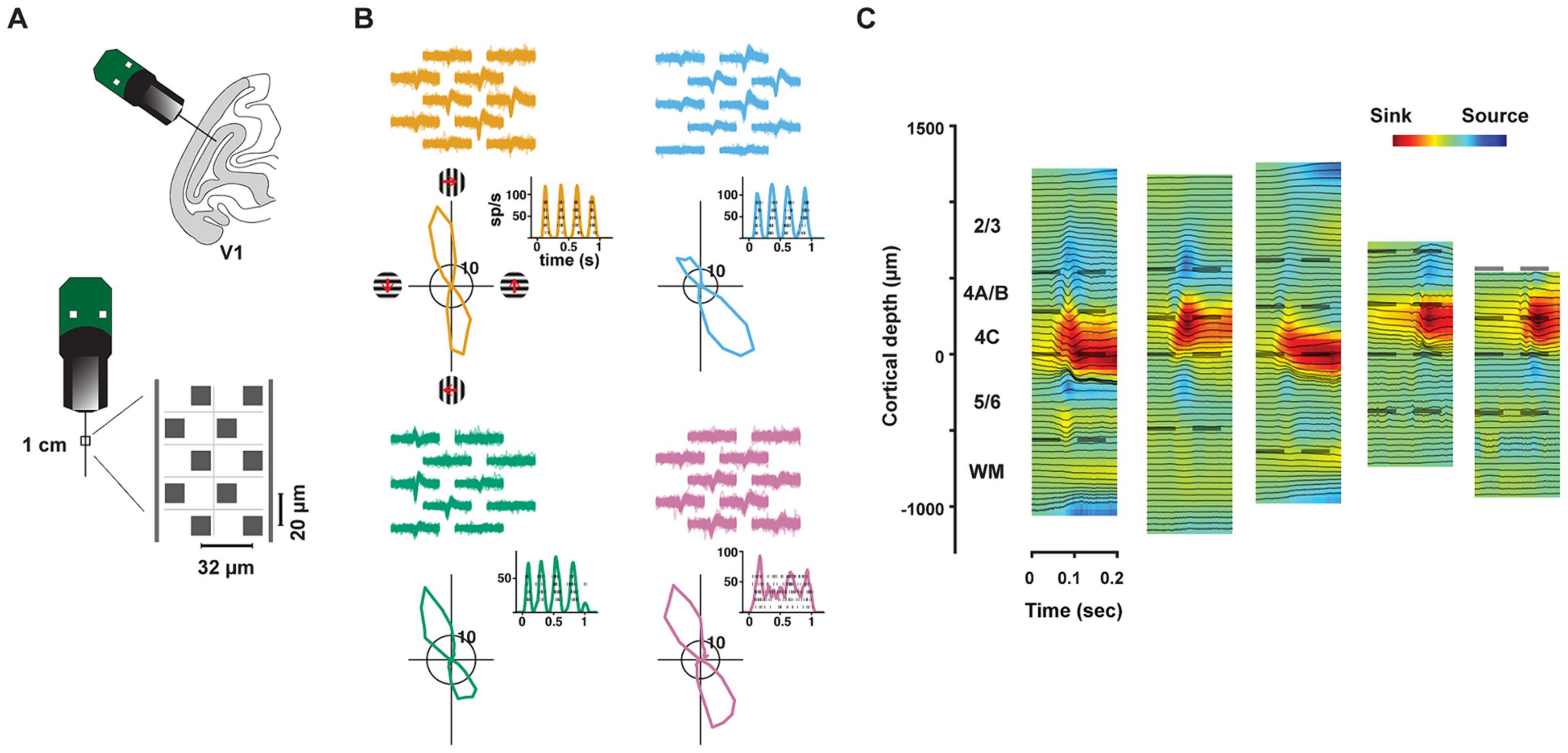
Neuropixels recordings in primate V1. **(A)** Upper cartoon depicts the angle of probe penetrations made into the lateral surface and underlying calcarine sulcus of V1. Lower, Image of Neuropixels probe base and shank. Right diagram shows the layout of electrode contacts for a section of the recording shank. **(B)** Example single-neuron recordings with Neuropixels probes, three simple cells (orange, blue, green) and 1 complex cell (purple). Top, neuronal waveforms recorded across multiple adjacent electrode contacts are shown for each neuron. Bottom, each neuron’s response to its preferred orientation (rasters and instantaneous spike rates) and their corresponding tuning curves. Red arrows denote the drift direction of oriented gratings. **(C)** CSD profiles for each of the five recording sessions. CSDs were derived from LFP responses to drifting gratings. In each session, laminar compartment boundaries (dashed lines) were determined using histological data and the CSD profile. WM: white matter.

We recorded the activity of neurons in V1 of two anesthetized rhesus macaques (*Macaca Mulatta*, M1, M2) using high-density, multi-contact Neuropixels probes (version 3A; IMEC Inc., Belgium) (Figure 1A) (methods). Each probe consisted of 986 contacts (12 μm x 12 μm, 20 μm spacing) distributed across 10 mm, of which 384 contacts could be simultaneously selected for recording. Probes were inserted into the lateral operculum of macaque V1 with the aid of a surgical microscope at angles nearly perpendicular to the cortical surface. Prior to probe insertion, it was coated with a DiI derivative for subsequent histological visualization of the probe track (methods). Recordings were made either in the opercular surface (M1) or within the underlying calcarine sulcus (M2) using the programmable channel selection of the Neuropixels probe (Supplementary Figure S1). The dense spacing between electrode contacts provided multiple measurements of the waveforms from individual neurons (mean = 4.52) and facilitated the isolation of each of a large number of single neurons (Figure 1B), typically >300 in a single penetration. In total, we recorded the activity of 1,833 well-isolated single neurons across layers of V1 in five penetrations in the two macaques (Sessions 1–3, M1: 1,124 neurons; Sessions 4–5, M2: 709 neurons). In each of the five recordings, we studied the functional properties of simultaneously recorded populations of neurons within different laminar compartments.

In order to assess the distribution of properties of V1 neurons across layers, we first estimated the borders of laminar compartments by combining the histological data with current-source density (CSD) measurements (Figure 1C) in each recording (methods). Using these estimates, we assigned each of the recorded neurons to a specific laminar compartment. Cortical layers were divided into four comparably sized laminar compartments, specifically, layers 2/3, 4A/B, 4C, and 5/6 (Mean thickness: 650 μm, 311 μm, 281 μm, 489 μm, respectively). We combined layers 4Cα and 4Cβ, the Magnocellular and Parvocellular recipient layers, respectively, into a single compartment in order to achieve comparable numbers of recorded neurons in each compartment. Notably, our compartmentalization of V1 layers was similar to previous studies relying on CSD profiles from linear arrays with larger contact spacing (Maier et al., 2011; Hansen et al., 2012; Self et al., 2013; Nandy et al., 2017; Wang et al., 2020; Gieselmann and Thiele, 2022). However, our approach leveraged the high density of Neuropixels probes combined with histology, providing finer resolution in laminar identification than most recent studies.

### Visual properties of simultaneously recorded neurons across V1 layers

A wealth of past electrophysiological studies has explored the differences in the functional properties of neurons across the layers of primate V1 (Schiller et al., 1976; Poggio et al., 1977; Bauer et al., 1980; Hawken and Parker, 1984; Livingstone and Hubel, 1984; Ringach et al., 2002; Gur et al., 2005). The most classically examined properties include firing rates, the proportions of simple and complex cells, the incidence of direction selectivity, and various components of orientation selectivity. The high-density recordings enabled us to assess these properties in the large numbers of visually responsive neurons recorded simultaneously in single sessions. Drifting Gabor gratings with a diameter of 1.5 degrees of visual angle (dva) were presented for 1 s within the joint receptive fields (RFs) of recorded neurons in 36 different directions (0–360°, 10° step). Four spatial frequencies (0.5, 1, 2, 4 cycles/deg) were tested and responses to the optimal spatial frequency were used in the analyses (methods). Six hundred and sixty one neurons (M1: 446; M2: 215) with responses during stimulus presentation period that were significantly different from the inter-stimulus interval (two-tailed *t*-test, *p* < 0.01) and elicited at least 3 spikes/s to the optimal stimulus conditions were included. In most of the recording sessions, data were obtained from >17 visually responsive neurons recorded in each laminar compartment (Supplementary Table S1).

**Figure 2:**
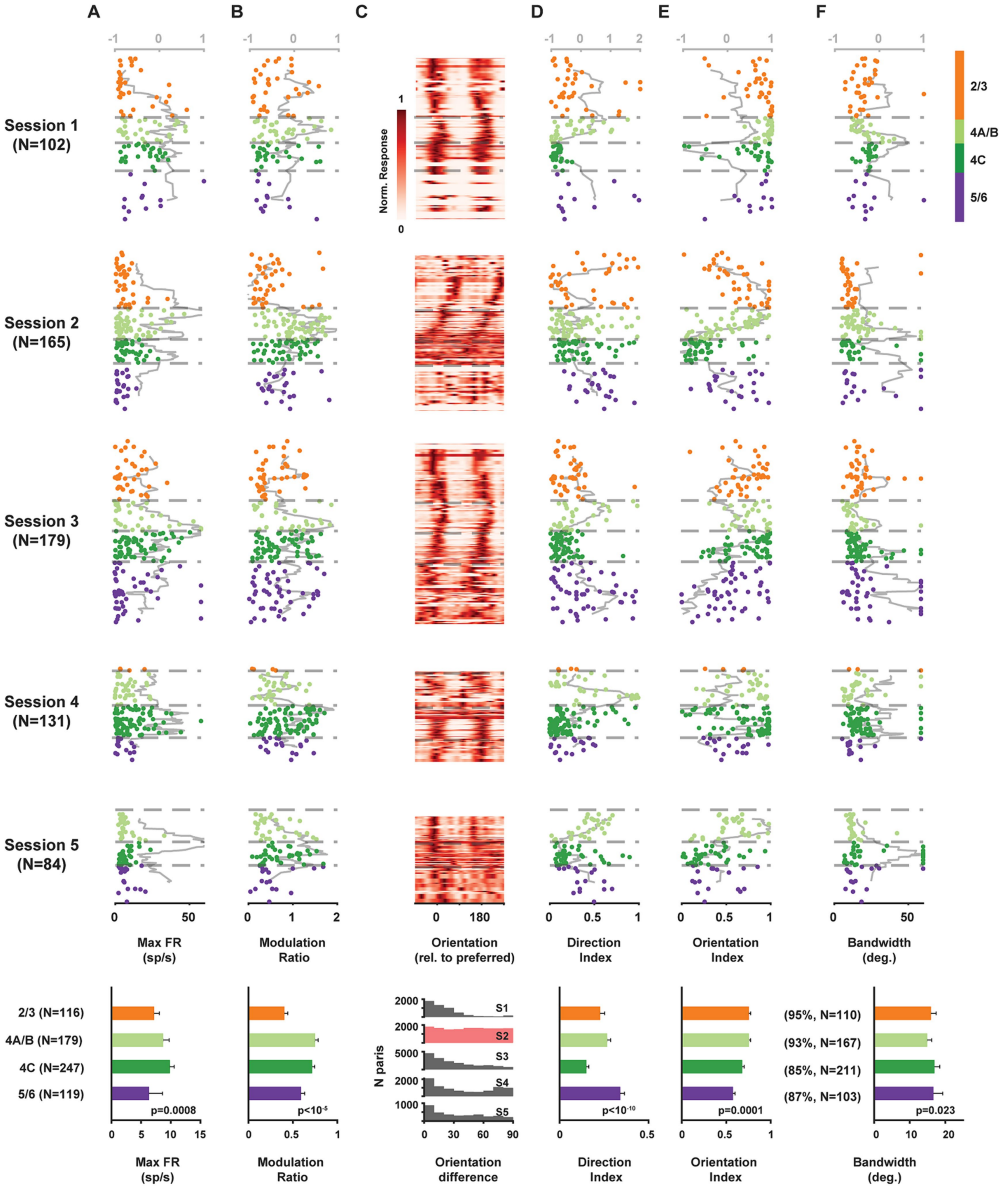
Functional properties of single V1 neurons recorded simultaneously across laminar compartments. Components of visual responses of all V1 neurons recorded in five sessions across identified laminar compartments. **(A)** Maximum firing rate response (to preferred stimulus). **(B)** Modulation ratio. **(C)** Heat map of visual responses across drift direction of oriented grating. Neurons are plotted at their relative cortical depth and stacked vertically. The vertical thickness for each neuron is adjusted based on local population density to minimize gaps while preserving depth accuracy, with greater thickness assigned to less dense neuronal populations. **(D)** Direction index. **(E)** Orientation index. **(F)** Orientation tuning bandwidth. Data from each neuron is plotted at its corresponding cortical depth. Gray lines (top abscissa) denote kurtosis of each value in a running 100 μm window. Bottom row plots show averaged results across all 5 sessions.

We found significant differences in the maximum firing rates of neurons located across laminar compartments (Kruskal-Wallis test, 𝛘^2^(3) = 16.65, *p* = 0.0008), with the highest median rates found in layer 4C (Figure 2A). Next, we compared the distribution of simple and complex cells across layers. Simple and complex cells are known to differ dramatically in their response to drifting gratings in that simple cells, being sensitive to phase, exhibit robust oscillatory modulation, while complex cells do not (De Valois et al., 1982) (Figure 1B). Thus, we used the modulation ratio of visual responses to reveal the differential distribution of simple and complex cells across layers in each recording (Figure 2B). As expected, modulation ratios varied significantly across laminar compartments (Kruskal-Wallis test, 𝛘^2^(3) = 28.55, *p* < 10^−5^), with larger ratios found among layer 4C neurons.

Given that our probe penetrations were made largely perpendicular to the cortical surface, we could visualize the known columnar organization of orientation tuning in V1 by simply plotting the orientation preference of individual neurons recorded across the cortical depth. Gratings drifted across all directions, and a majority of neurons exhibited peak responses at two orientations that were equal but moving in opposite directions (Figure 2C). Moreover, for most recordings (sessions 1, 3–5), the preferred orientation remained similar for neurons distributed across depth, indicating that these penetrations remained largely within a single orientation column (*columnar* sessions). In contrast, the preferred orientation varied systematically across cortical depth in session 2 (*non-columnar* session). Across recordings, a subset of neurons responded more strongly to one of the drift directions, thus exhibiting direction selectivity. Direction selectivity was quantified using a standard selectivity index that compared the preferred drift direction to the opposite direction (methods) (Figure 2D). Direction selectivity varied significantly across layers (Kruskal-Wallis test, 𝛘^2^(3) = 52.36, *p* < 10^−10^), with significantly lower values in layer 4C (two-tailed Wilcoxon rank-sum test, *p* < 10^−10^). Similarly, for each neuron, we also computed a selectivity index for orientation, by comparing responses to the preferred and orthogonal orientations (methods) (Figure 2E). As with direction selectivity, orientation selectivity varied significantly across layers (Kruskal-Wallis test, 𝛘^2^(3) = 20.33, *p* = 0.0001). However, consistent with previous studies (Schiller et al., 1976; Hawken and Parker, 1984; Ringach et al., 2002), orientation selectivity was not significantly lower in layer 4C than in other layers (all sessions: *n_4C_* = 247, *n_others_* = 414, Median: 4C 0.68, others 0.70, *p* = 0.084; all columnar sessions: Median: 4C 0.77, others 0.72, *p* = 0.52; two-tailed Wilcoxon rank-sum test). For most of the recorded neurons, responses across orientations were well fit by a circular Gaussian (median *R^2^* = 0.95) (methods). From the population of well-fit neurons (*R^2^* ≥ 0.7, 89.4%), we obtained tuning bandwidths and compared them across cortical depth (Figures 2F). Overall, tuning bandwidths differed significantly across layers (Kruskal-Wallis test, 𝛘^2^(3) = 9.51, *p* = 0.023), consistent with previous evidence (Ringach et al., 2002). Bandwidth was very slightly, but significantly, greater in 4C compared to other layers (All sessions: *n_4C_* = 211, *n_others_* = 380, Median: 4C 17.0, others 15.7, *p* = 0.012; All columnar sessions: Median: 4C 17.5, others 16.5, *p* = 0.011; two-tailed Wilcoxon rank-sum test).

### Superior decoding of orientation from layer 4 neurons

Orientation selectivity emerges within primate V1 and is thus perhaps the most fundamental property of primate V1 neurons. Differences in orientation selectivity of neurons within different layers have been the focus of numerous previous studies (Schiller et al., 1976; Poggio et al., 1977; Bauer et al., 1980; Hawken and Parker, 1984; Livingstone and Hubel, 1984; Ringach et al., 1997; Ringach et al., 2002; Gur et al., 2005). Similar to our observations, these studies found equivocal differences in the orientation selectivity of individual neurons distributed across layers. However, classical measurements of selectivity (e.g., bandwidth, selectivity index) are limited in their ability to adequately quantify the information contained in the responses of sensory neurons. As an alternative, more recent studies have deployed machine learning algorithms to decode orientation signals contained in the responses of populations of simultaneously recorded V1 neurons (Graf et al., 2011; Berens et al., 2012). These studies captured the rapid and highly orientation-sensitive signals conveyed by populations of V1 neurons. However, recordings in these studies did not allow for simultaneous comparisons of orientation decoding between different subpopulations of neurons within layers of the same cortical column. Thus, we leveraged our high-density recordings to examine the strength of orientation signals within different V1 layers using a decoding approach. Using the same data set, we employed five commonly used machine learning algorithms to predict visual stimuli based on the activity of neuronal subpopulations recorded simultaneously across V1 layers. Specifically, the algorithms included Logistic Regression, Linear Discriminant Analysis, Support Vector Machine, Random Forest, and the correlation-blind classifier Gaussian Naïve Bayes. The chosen algorithms encompassed a diverse set of complementary approaches based on different decoding principles (methods).

**Figure 3:**
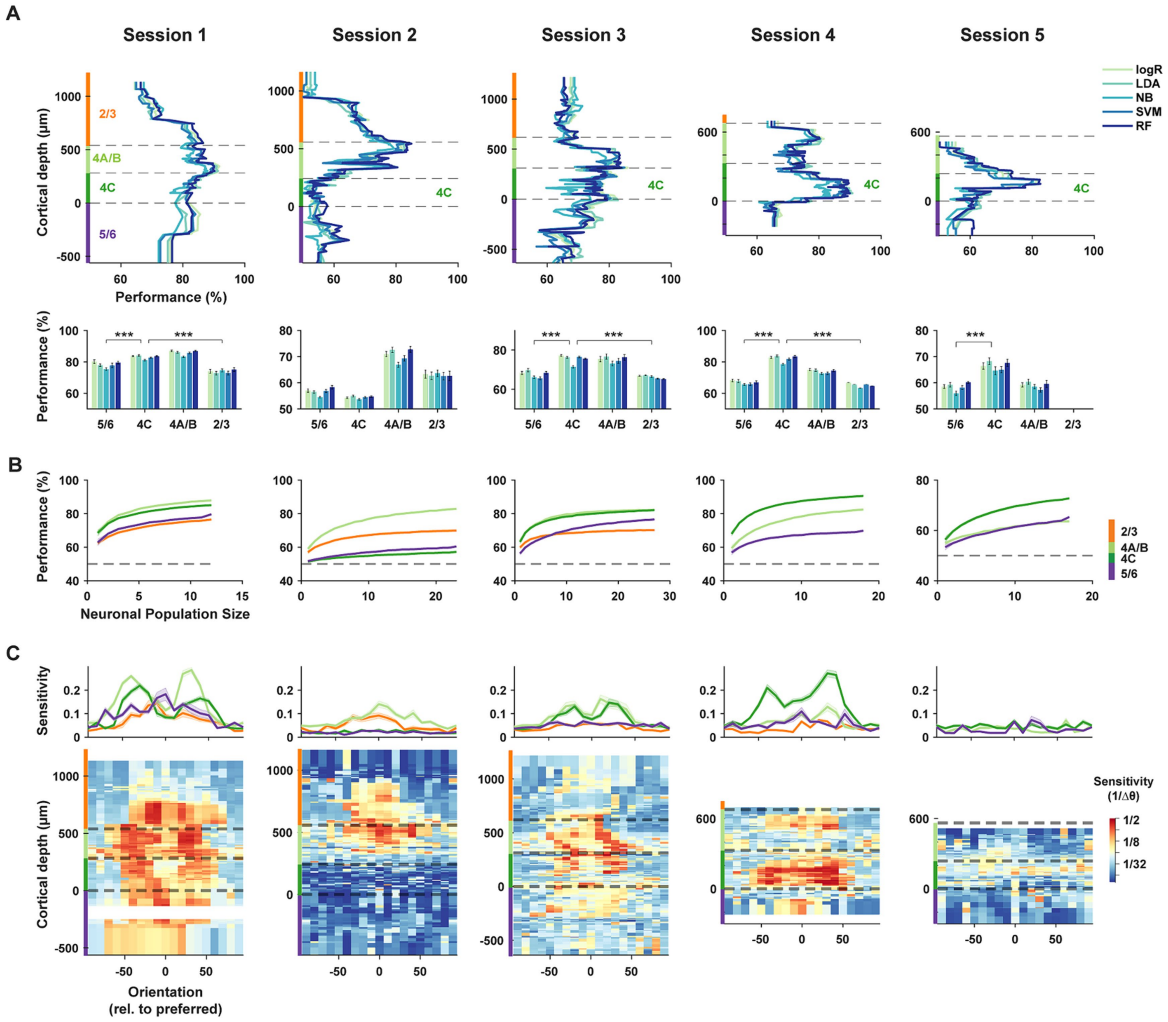
Decoding of orientation from laminar subpopulations of V1 neurons. **(A)** Top, orientation decoding performance using five complementary machine learning algorithms for laminar subpopulations within each recording session. LogR: Logistic Regression; LDA: Linear Discriminant Analysis; NB: Gaussian Naïve Bayes; SVM: Support Vector Machine; RF: Random Forest. Decoders were trained on responses from 10 adjacent neurons within different laminar compartments, and performance was averaged across all pairwise orientation discriminations. Dashed lines indicate the cortical depth of laminar compartment boundaries, with different compartments highlighted by color bars on the ordinate axis. Bottom, mean decoding accuracy for each algorithm, averaged across subpopulations within each laminar compartment. ***p < 10^−3^. **(B)** Neuron dropping curves. Performance of the LDA algorithm for subpopulations of varying size from different laminar compartments. **(C)** Sensitivity of orientation decoding across cortical depth. Sensitivity is defined as the reciprocal of the minimum orientation change required to achieve 60% accuracy using the LDA algorithm. Each recording session is aligned to the preferred orientation of the neuronal population. Top, average sensitivities across subpopulations from different laminar compartments. Error bars denote ± S.E.M.

Using data from each neuronal subpopulation recorded during a session, we evaluated the accuracy of each decoding algorithm in discriminating between pairs of stimulus orientations (methods). We assessed pairwise discrimination performance for all 153 combinations of the 18 tested orientations, using responses from a constant number of neurons (*n* = 10) within each laminar subpopulation. These pairwise discrimination performances were then averaged for each subpopulation (Figure 3A). While the five algorithms generally performed similarly, distinct differences were evident among neuronal subpopulations distributed across cortical depths. Specifically, we found that subpopulations of layer 4C neurons consistently outperformed subpopulations within layers 2/3 and 5/6. Only in the non-columnar session (session 2), in which the preferred orientation varied widely across cortical depth (Figure 2C), did the performance of layer 4C decoders fall short compared to that of other layers. When compared to other laminar subpopulations, the average decoding performance of layer 4C decoders across the five algorithms in columnar sessions (sessions 1, 3–5) significantly exceeded that of superficial (L2/3) and deep (L5/6) layers (4C vs. 2/3: *Δ*% = [9.1, −8.5, 9.2, 16.9], *p* = [2.5*10^−6^, 6.2*10^−3^, 3.3*10^−17^, 8.2*10^−4^], for sessions 1–4 respectively; 4C vs. 5/6: Δ% = [4.8, −2.2, 7.8, 15.2, 8.0], *p* = [4.9*10^−4^, 2.8*10^−4^, 1.8*10^−12^, 4.1*10^−10^, 6.1*10^−4^], for sessions 1–5 respectively; two-tailed Wilcoxon rank-sum test). Decoding performance for layer 4A/B neurons also exceeded that of superficial (L2/3) and deep (L5/6) layers for the majority of the sessions (4A/B vs. 2/3: Δ% = [11.9, 7.6, 9.0, 8.7], *p* = [7.9*10^−10^, 2.3*10^−4^, 7.2*10^−6^, 2.6*10^−3^], for sessions 1–4 respectively; 4A/B vs. 5/6: Δ% = [7.6, 13.9, 7.6, 7.0, 0.6], *p* = [1.8*10^−6^, 8.3*10^−11^, 4.8*10^−5^, 1.3*10^−5^, 0.9], for sessions 1–5 respectively; two-tailed Wilcoxon rank-sum test).

We considered that the apparent superiority of layer 4 neurons (4A/B and 4C) at discriminating orientation could have resulted from the arbitrary number of neurons chosen (*n* = 10) in each neuronal subset. Thus, for each session, we also generated neuron-dropping curves (NDCs) (Wessberg et al., 2000) from the performance of neuronal subsets obtained in each laminar compartment in order to compare performance across varying population sizes (Figure 3B). For each of the columnar sessions (sessions 1, 3–5), the NDC revealed greater performance for layer 4 neurons across the range of population sizes compared to superficial (L2/3) and deep (L5/6) layers.

Next, we measured the sensitivity of laminar subpopulations to orientation changes across the range of orientations. For each orientation, sensitivity was measured as the reciprocal of the threshold change in orientation required to exceed 60% performance in neuronal subpopulations distributed across layers (methods). Peaks in orientation discrimination sensitivity were typically observed on the flanks of the preferred orientation of the constituent neurons (Figure 3C) corresponding to the steepest points in the orientation tuning curves, especially for layer 4C neurons. Across sessions, sensitivity was consistently highest in middle layers 4A/B and 4C, with the lowest values found in the 5/6 compartment. Thus, in the columnar recordings, populations of layer 4 neurons exhibited greater orientation sensitivity than their superficial and deep layer counterparts.

### Single neuron properties contribute to superior orientation decoding in layer 4C

We next considered the extent to which the superior decoding of orientation in layer 4 might be due to the reduced correlated variability there. Much experimental and theoretical work describes how noise correlations can reduce or limit the amount of information available in the responses of neuronal populations (Abbott and Dayan, 1999; Averbeck et al., 2006; Cohen and Kohn, 2011). Indeed, superior orientation discrimination in layer 4 was previously predicted from the observation of reduced correlated variability (Hansen et al., 2012). However, superior layer 4 performance appeared to be present in the correlation-blind decoders (Gaussian Naïve Bayes), and even in very small populations or single neurons (NDCs, Figure 3B), suggesting that correlated variability was not a key factor.

**Figure 4:**
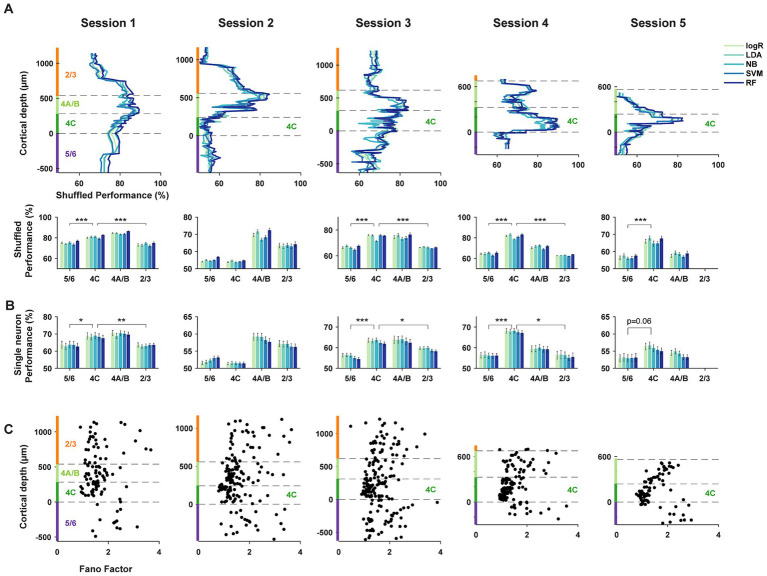
Trial-shuffled and single-neuron decoding of orientation. **(A)** Decoding performance as in Figure 3A, but using trial-shuffled data. Trials from different neurons within each subpopulation were shuffled independently. ***p < 10^−3^. **(B)** Decoding performance as in Figure 3A, but using responses of single neurons. ***p < 10^−3^, **p < 10^−2^, *p < 0.05. **(C)** Fano factors for single neurons across different laminar compartments.

To address this more directly, we repeated the decoding comparisons using shuffled trials, thus largely removing correlated activity (methods). Comparisons of the discrimination performance between shuffled and unshuffled datasets revealed no differences, or very small differences, across laminar compartments (2/3: *Δ*% = 0.05 ± 0.12, *p* = 0.62; 4A/B: Δ% = −1.48 ± 0.11, *p* = 1.4*10^−26^; 4C: Δ% = −0.74 ± 0.07, *p* = 1.0*10^−20^; 5/6: Δ% = −2.10 ± 0.16, *p* = 1.0*10^−19^. Shuffled-unshuffled, mean ± S.E.M, two-tailed Wilcoxon signed-rank test). As with the unshuffled datasets, decoders trained on the activity of shuffled populations also exhibited clear differences in orientation discrimination across different laminar compartments. In the trial shuffled populations, populations of layer 4 neurons consistently outperformed populations in layers 2/3 and 5/6 in columnar recordings (Figure 4A). When compared to other laminar subpopulations, the average decoding performance across the five algorithms of layer 4C neurons in columnar sessions (sessions 1, 3–5) significantly exceeded that of superficial (L2/3) and deep (L5/6) layers (4C vs. 2/3: *Δ*% = [7.3, −9.3, 8.5, 18.6], *p* = [8.8*10^−6^, 1.3*10^−4^, 4.5*10^−17^, 8.2*10^−4^], for sessions 1–4 respectively; 4C vs. 5/6: Δ% = [5.8, −0.9, 8.4, 17.0, 9.4], *p* = [2.5*10^−5^, 4.6*10^−3^, 1.8*10^−14^, 1.5*10^−10^, 5.7*10^−5^], for sessions 1–5 respectively; two-tailed Wilcoxon rank-sum test). Decoding performance for layer 4A/B neurons also exceeded that of superficial (L2/3) and deep (L5/6) layers for the majority of the sessions (4A/B vs. 2/3: Δ% = [11.0, 6.5, 8.4, 8.1], *p* = [1.9*10^−9^, 5.4*10^−4^, 1.4*10^−5^, 2.2*10^−3^], for sessions 1–4 respectively; 4A/B vs. 5/6: Δ% = [9.5, 14.9, 8.3, 6.5, 1.5], *p* = [5.8*10^−7^, 5.2*10^−12^, 8.8*10^−6^, 5.5*10^−6^, 0.4], for sessions 1–5 respectively; two-tailed Wilcoxon rank-sum test).

Given that single-neuron properties seemed to be contributing more to differences in decoding performance, we used the same decoders to discriminate orientation from the activity of single neurons within different laminar compartments (Figure 4B) (methods). As expected, the overall average performance of single-neuron decoders at discriminating orientation pairs was reduced compared to that of 10-neuron subpopulations. However, the pattern of results was remarkably similar between the single neuron and population-level analyses. Specifically, in columnar sessions (sessions 1, 3–5), the average decoding performance across the five algorithms of layer 4C single-neurons significantly exceeded that of superficial (L2/3) and deep (L5/6) layers (4C vs. 2/3: Δ% = [5.1, −5.3, 3.7, 11.8], *p* = [3.3*10^−3^, 1.5*10^−5^, 0.014, 0.025], for sessions 1–4 respectively; 4C vs. 5/6: Δ% = [5.0, −0.9, 7.2, 11.6, 2.8], *p* = [0.034, 0.13, 3.6*10^−6^, 1.3*10^−5^, 0.06], for sessions 1–5 respectively; two-tailed Wilcoxon rank-sum test). The decoding performance for layer 4A/B neurons also showed a trend to exceed that of superficial (L2/3) and deep (L5/6) layers (4A/B vs. 2/3: Δ% = [6.7, 1.9, 4.2, 3.5], *p* = [2.5*10^−3^, 0.31, 0.048, 0.57], for sessions 1–4 respectively; 4A/B vs. 5/6: Δ% = [6.6, 6.3, 7.7, 3.3, 1.0], *p* = [0.025, 2.1*10^−4^, 2.1*10^−4^, 0.27, 0.32], for sessions 1–5 respectively; two-tailed Wilcoxon rank-sum test), similar to the population-level comparisons. Thus, the properties of single neurons were sufficient to yield superior performance of layer 4 decoders. We observed a similar pattern of results when comparing performance using a multi-class decoder and across spatial frequency (Supplementary Figure S2).

Given the lack of substantial differences in the basic tuning measures between neurons across layers, e.g., orientation index and tuning bandwidth, it is surprising that single neuron decoding of layer 4 neurons exceeded that of superficial and deep neurons. However, these measures fail to fully capture the information available in sensory responses. In particular, these measures do not account for differences in the reliability of stimulus-driven responses between different neurons, for example differences in the Fano factor (FF) (Churchland et al., 2006; Churchland et al., 2010; Steinmetz and Moore, 2010). Thus, we considered the possibility that layer 4C neuronal responses might be more reliable than those of superficial and deep neurons. Previous studies comparing the FFs of V1 neurons across layers yielded equivocal results (Hansen et al., 2012; Gur and Snodderly, 2006), perhaps due to comparatively small datasets. We compared the FFs of neurons within the different laminar compartments (methods) (Figure 4C). We found highly robust differences in the FF of visual responses across laminar compartments (2/3 (*N* = 116): 1.87 ± 0.07, 4A/B (*N* = 179): 1.62 ± 0.04, 4C (*N* = 247): 1.28 ± 0.02, 5/6 (*N* = 119): 1.94 ± 0.06; Kruskal-Wallis test, 𝛘^2^(3) =167.32, *p* = 4.8*10^−36^). Moreover, the FFs of layer 4C neurons were significantly lower than that of neurons in layers 2/3 (*p* = 5.2*10^−22^, two-tailed Wilcoxon signed-rank test), 5/6 (*p* = 3.5*10^−23^), and also 4A/B (*p* = 4.2*10^−19^). The Fano factors of layers 4A/B neurons were also significantly lower than that of neurons in layers 2/3 (*p* = 4.3*10^−3^), 5/6 (*p* = 6.3*10^−5^). These differences were not a result of differences in firing rate across layers (Supplementary Figure S3A) and were present across all stimulus orientations (Supplementary Figure S3B). Across the full population, the performance of 10-neuron subpopulation decoders (Figure 3A) was negatively correlated with the mean FF of the comprising neurons (*r* = −0.32, *p* = 1.5*10^−17^). The performance of single-neuron decoders (Figure 4B) was also negatively correlated with the FF of the corresponding single neurons (*r* = −0.09; *p* = 0.014). Thus, the greater performance of layer 4C neurons was associated with larger reliability in single neuron responses. Given that the orientation selectivity indices and tuning bandwidths of layer 4C neurons were largely comparable to those in other layers (Figures 2E,F), it is likely that the superior orientation decoding in 4C is at least partly due to the greater response reliability of 4C neurons.

## Discussion

We studied the visual activity of large populations of neurons distributed across layers in primate V1 using high-density Neuropixels probes. The high capacity of the probes enabled single-neuron recordings from a substantial number of nearby cells within the same laminar compartments of single cortical columns, thereby facilitating robust comparisons between different subpopulations of neurons within single experiments. Our comparisons revealed myriad differences in the functional properties of neurons across layers, including higher firing rates but lower proportions of complex or direction-selective neurons in layer 4C. Despite extensive prior studies, standard measures of orientation selectivity across layers yielded equivocal results regarding clear differences between layers. To address this, we employed a decoding approach to evaluate the orientation discrimination performance achievable from the activity of subpopulations within different laminar compartments.

Previous studies have examined a number of factors that could affect the decoding of sensory information in the cortex, such as single-neuron selectivity indices, bandwidth, response strength (Vogels and Orban, 1990; Kang et al., 2004), tuning preferences (Jazayeri and Movshon, 2006), as well as both signal and noise correlation (Graf et al., 2011; Berens et al., 2012; Panzeri et al., 2022). While some studies have highlighted the importance of response reliability (Vogels and Orban, 1990; Butts and Goldman, 2006; Montijn et al., 2014; Nogueira et al., 2020), less is known about whether and how these factors differ in a layer-dependent manner.

Surprisingly, we found that in columnar recording sessions, decoder performance in layer 4C was superior to that of superficial and deep layers. This is the first observation of an unambiguous difference in orientation discrimination between neurons spanning different layers of the same V1 column. Importantly, the superior orientation discrimination from layer 4C was not dependent on differences in correlated variability observed between laminar compartments, as the same pattern was observed in both the correlation-blind decoder and in the trial-shuffled datasets. In addition, single-neuron decoding yielded an identical pattern of results, with orientation decoding from layer 4C neurons exceeding the performance of superficial and deep layer neurons. Instead, the superior orientation discrimination was associated with reduced response variability in layer 4C neuronal responses. This result not only contrasts with the classic view that orientation selectivity is largely absent among neurons in layer 4C of primate V1, particularly 4Cβ (Livingstone and Hubel, 1984; Blasdel and Fitzpatrick, 1984), and confirms earlier evidence of clear orientation tuning in 4C (Schiller et al., 1976; Hawken and Parker, 1984; Ringach et al., 2002; Gur et al., 2005; Wang et al., 2020), but it also demonstrates that when comparing neurons within the same column, the fidelity of orientation information is at its peak in the output of layer 4C neurons.

Importantly, our finding of superior orientation decoding in layer 4C does not necessarily suggest that layer 4C entirely derives its orientation selectivity from subcortical inputs. Although previous studies have demonstrated an orientation bias in the thalamic input to V1, particularly in rodents (Priebe, 2016), it is generally believed that primate LGN input to V1 columns is sparse and only weakly orientation-tuned. Instead, primate V1 layer 4C neurons are thought to amplify and sharpen the orientation selectivity through a combination of spatial alignment of thalamic input and local intracortical recurrent excitatory and inhibitory connections (Chariker et al., 2016). Once orientation selectivity emerges in this layer, it is transferred to other layers in a columnar fashion through interlaminar relays, where further processing occurs (Lund et al., 2003).

Although our experiments were performed in anesthetized macaques, our findings are likely generalizable to awake animals. For example, our findings of clear orientation tuning in layer 4C align well with previous reports in both awake monkeys (Gur et al., 2005, Wang et al., 2020) and anesthetized monkeys (Schiller et al., 1976; Hawken and Parker, 1984; Ringach et al., 2002). The lower response variability we found in individual layer 4C neurons is also consistent with the lower correlated variability (noise correlation) reported previously in both awake (Hansen et al., 2012) and anesthetized monkeys (Smith et al., 2013). In the study of awake monkey, a tendency of neurons in granular layer (layer 4) to exhibit lower response variability was also noted (Hansen et al., 2012). Additionally, they reported superior orientation decoding in layer 4, attributing this to reduced shared variability. Our current work expands upon these findings by demonstrating that, even when disrupting the correlated variability within the dataset or using correlation-blind decoders, layer 4 neurons consistently outperformed superficial and deep layers.

We found that sensitivity for orientation discrimination was typically highest at the flanks of the preferred orientation of the constituent neurons (Figure 3C), corresponding to the steepest points in the orientation tuning curve, especially for layer 4C neurons in sessions 1, 3, and 4. In contrast, the highest sensitivity for layers 5/6 neurons typically occurred at the peak points in the orientation tuning curve. Given that layer 4C neurons exhibited lower response variability than layers 5/6 neurons, this aligns well with previous findings showing that in orientation discrimination, the best encoded stimulus can transition from high-slope to high-firing-rate regions of the tuning curve as the noise level in neuronal responses increases (Butts and Goldman, 2006).

The layer dependence of response variability we observed could involve different underlying circuits. Different V1 layers are involved in different feedforward and feedback stages of visual processing, which involve distinct intra- and inter-cortical connections. Layer 4C is the main feedforward input layer, which is driven predominantly by input from LGN (Blasdel and Lund, 1983); whereas feedback connections from higher areas and horizontal connections from within V1 principally target superficial and deep layers, largely avoiding layer 4C (Rockland and Lund, 1983; Rockland and Pandya, 1979; Rockland and Virga, 1989; Markov et al., 2014). Previous studies have shown that response variability increases from retina to cortex (Kara et al., 2000). Therefore, neurons in layer 4C, particularly simple cells receiving reliable synaptic input from the LGN, May exhibit lower variability compared to neurons in more superficial and deeper layers that receive more feedback/horizontal inputs (Movshon, 2000). Consistent with this notion, we observed a negative correlation between Fano factor and the simple/complex modulation index (*r* = −0.28, *p* = 2.2*10^−13^). That is, simple cells, which are more prevalent in layer 4C, tend to display smaller response variability. Another potential contributor to laminar differences in response variability could arise from different levels of inhibition across layers. Previous studies suggest that neural variability is tightly controlled by inhibitory networks, with increased inhibition associated with reduced response variability (Haider et al., 2010; Thiele et al., 2012). Suppression of parvalbumin (PV) interneuron activity leads to larger response variability in both anesthetized and awake animals (Zhu et al., 2015). Previous anatomical studies demonstrate that PV neurons are more prevalent in layers 2–4, especially layer 4C in Macaque V1 (Van Brederode et al., 1990; Kelly et al., 2019; Kooijmans et al., 2020). Thus, it is plausible that layer 4C activity involves increased inhibitory input from local PV interneurons, contributing to lower response variability.

The visual system of the macaque monkey has proven to be remarkably similar to that of the human and is thus an ideal model. However, in contrast to simpler model systems, extracting circuit-level information from studies of the nonhuman primate visual system has proven particularly challenging given the limited arsenal of appropriate tools. One key shortcoming of past neurophysiological studies in nonhuman primates is their relative inability to capture the diversity of neural signals present within both local and distributed populations of neurons in simultaneous recordings. Neurophysiological studies within the primate visual system have largely involved successive recordings from individual neurons, or small numbers of neurons using conventional single-electrodes, or low-channel count linear arrays. From such data, neuronal properties are studied in aggregated datasets of recordings accumulated across multiple sessions. As a result, direct comparisons between subpopulations of neurons within local circuits, e.g., within single cortical columns, are less than ideal. Although many studies employing implanted arrays have yielded datasets from ~100 s simultaneously recorded neurons, particularly within the motor system (Wannig et al., 2011; Churchland et al., 2012), such recordings can only be achieved within surface (and flat) cortical areas, and importantly, tend to restrict sampling of neurons at a fixed depth. A number of recent studies have demonstrated the advantages of recently developed high-density silicon probes, particularly Neuropixels probes, in capturing the properties and dynamics of large populations of local and distributed neurons (Jun et al., 2017; Steinmetz et al., 2019; Steinmetz et al., 2018; Trepka et al., 2022; Sun et al., 2022; Trautmann et al., 2023). In these first high-density recordings of primate V1, we have shown the value of such an approach in revealing the major properties of neurons comprising neocortical columns, a fundamental unit of neocortical circuitry.

## Data Availability

The raw data supporting the conclusions of this article will be made available by the authors, without undue reservation.

## Footnotes

^1^https://github.com/cortex-lab/phy
