# Supplementary material for: Comparison of orientation encoding across layers within single columns of primate V1 revealed by high-density recordings
Source: Front Neural Circuits. 2024 Sep 23;18:1399571. doi: 10.3389/fncir.2024.1399571 (PMC11456443; doi:10.3389/fncir.2024.1399571)
Supplement: Supplementary file 1 [file Table_1.DOCX]

***Supplementary table 1***

Counts of visual responsive neurons:

|  | Session1 | Session2 | Session3 | Session4 | Session5 | Mean |
| --- | --- | --- | --- | --- | --- | --- |
| 2/3 | 33 | 40 | 40 | 4 |  | 37.7 |
| 4A/B | 30 | 57 | 29 | 33 | 30 | 35.8 |
| 4C | 27 | 46 | 61 | 76 | 37 | 49.4 |
| 5/6 | 12 | 23 | 49 | 18 | 17 | 23.8 |
