# Supplementary figures and images for: Comparison of orientation encoding across layers within single columns of primate V1 revealed by high-density recordings
Source: Front Neural Circuits. 2024 Sep 23;18:1399571. doi: 10.3389/fncir.2024.1399571 (PMC11456443; doi:10.3389/fncir.2024.1399571)

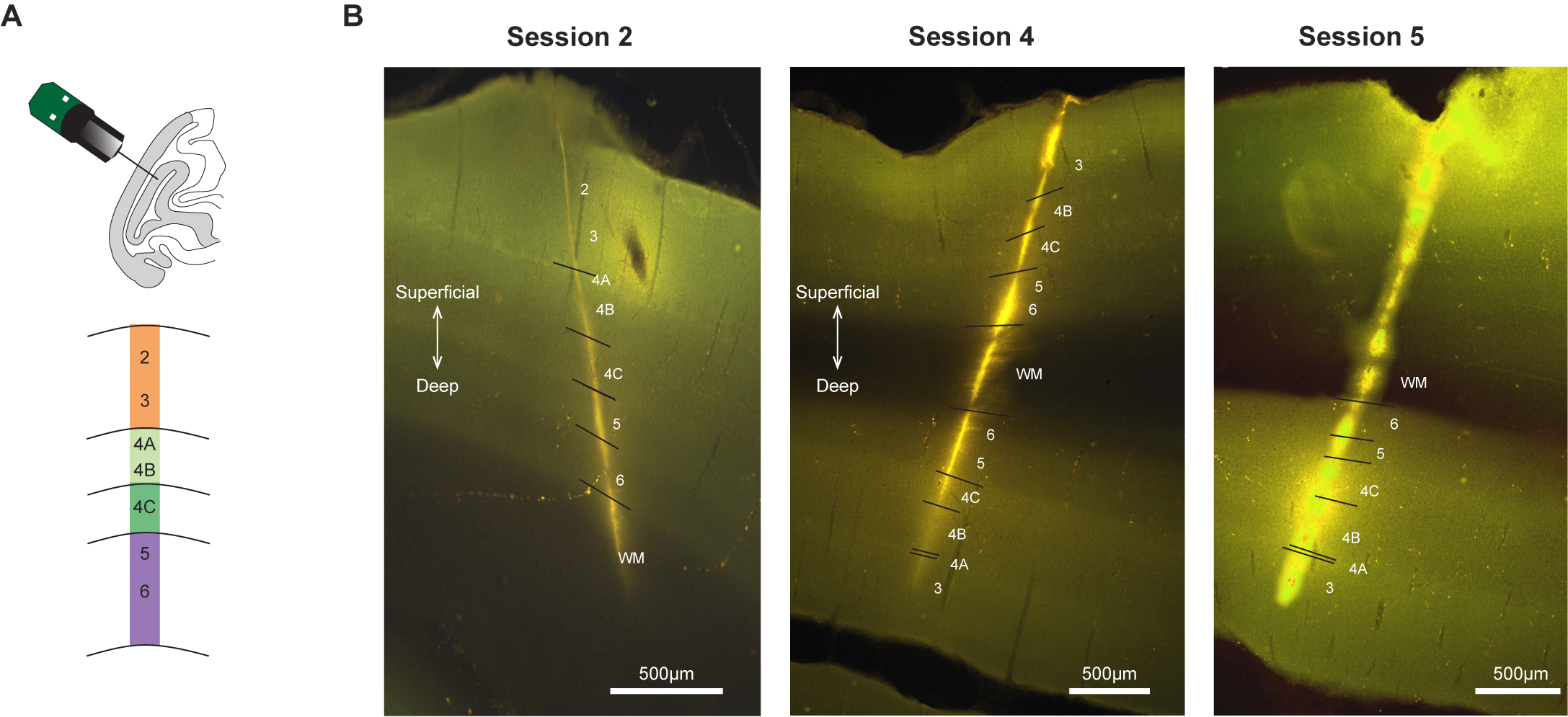

Supplement: Supplementary file 2 [file Image_1.TIF]

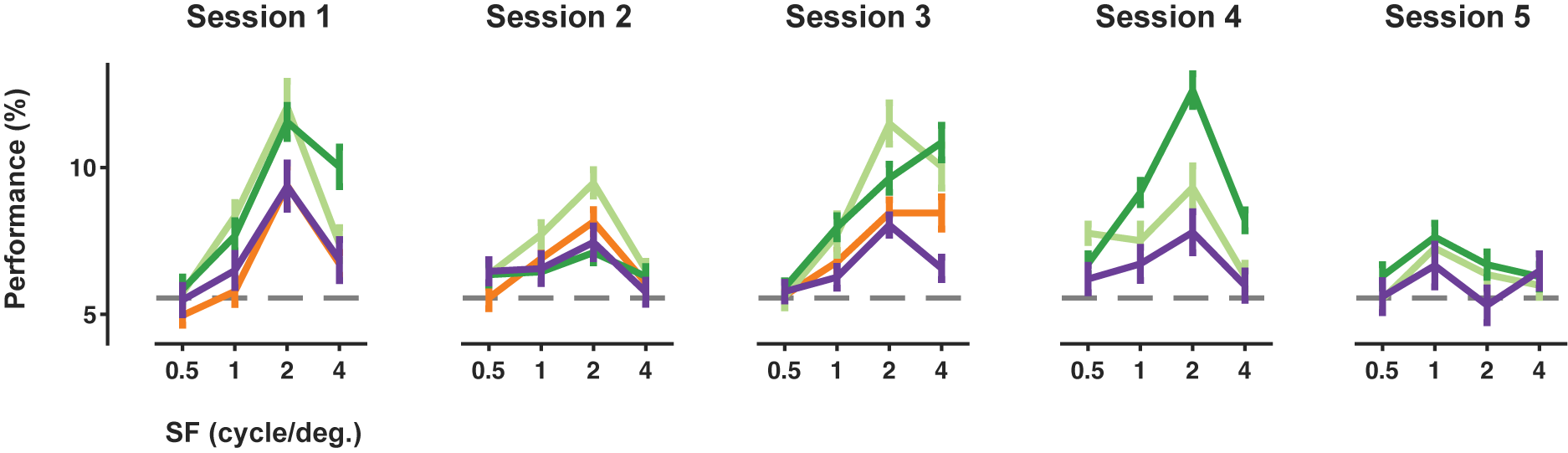

Supplement: Supplementary file 3 [file Image_2.TIF]

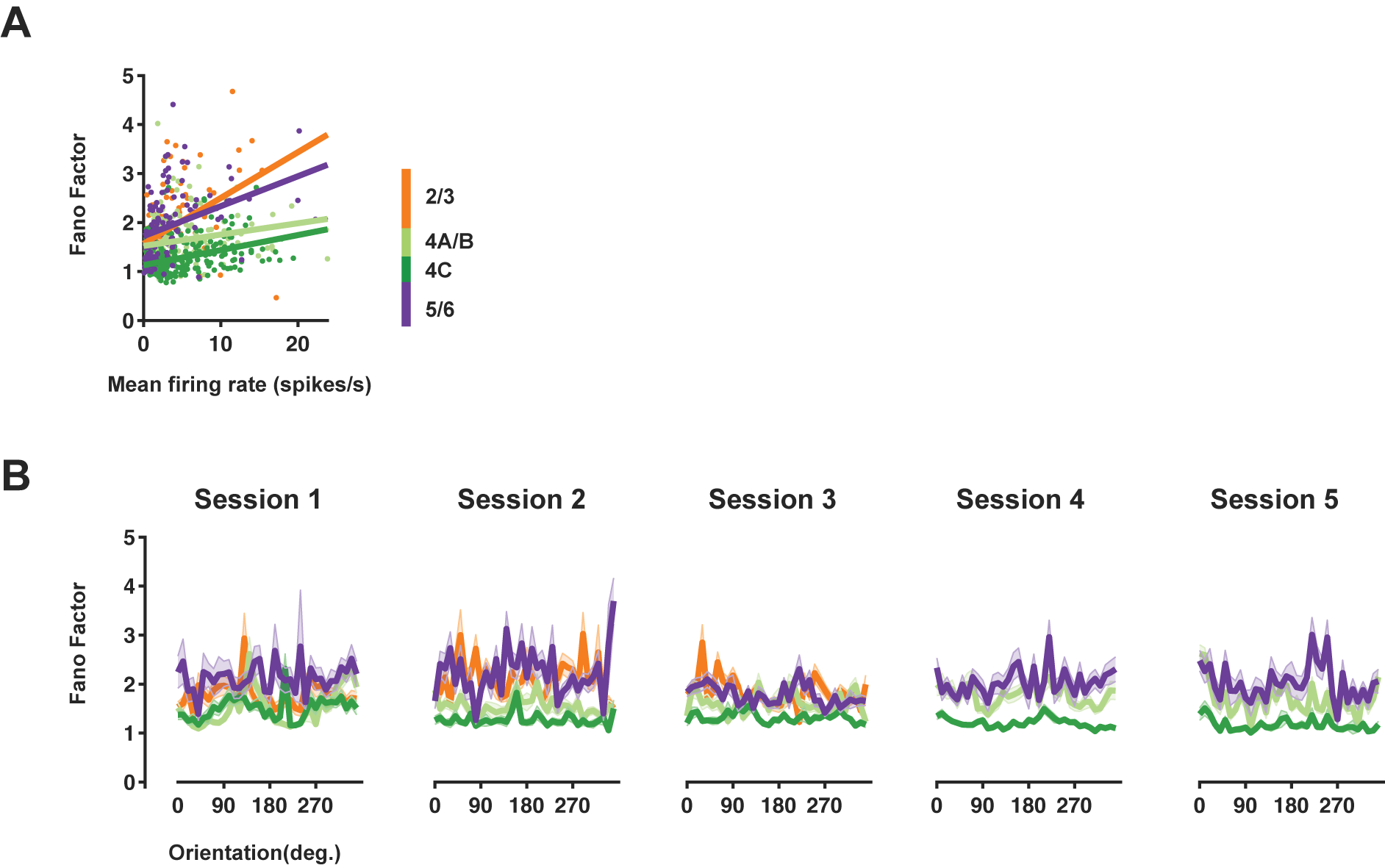

Supplement: Supplementary file 4 [file Image_3.TIF]
